# Adenosine Deaminase Enhances the Immunogenicity of Human Dendritic Cells from Healthy and HIV-Infected Individuals

**DOI:** 10.1371/journal.pone.0051287

**Published:** 2012-12-11

**Authors:** Víctor Casanova, Isaac Naval-Macabuhay, Marta Massanella, Marta Rodríguez-García, Julià Blanco, José M. Gatell, Felipe García, Teresa Gallart, Carme Lluis, Josefa Mallol, Rafael Franco, Núria Climent, Peter J. McCormick

**Affiliations:** 1 Department of Biochemistry and Molecular Biology, Faculty of Biology, University of Barcelona, Barcelona, Spain; 2 Fundació irsiCaixa-HIVACAT, Institut d’Investigació en Ciències de la Salut Germans Trias i Pujol, Badalona, Spain; 3 Department of Physiology and Neurobiology, Dartmouth Medical School Lebanon, Lebanon, New Hampshire, United States of America; 4 Institut d'Investigacions Biomèdiques August Pi i Sunyer (IDIBAPS)-AIDS Research Group, and Catalonian Center for HIV Vaccines (HIVACAT), Barcelona, Spain; 5 Infectious Diseases and AIDS Unit, Hospital Clínic de Barcelona, Barcelona, Spain; 6 Service of Immunology, Hospital Clínic de Barcelona, Barcelona, Spain; South Texas Veterans Health Care System and University Health Science Center San Antonio, United States of America

## Abstract

ADA is an enzyme implicated in purine metabolism, and is critical to ensure normal immune function. Its congenital deficit leads to severe combined immunodeficiency (SCID). ADA binding to adenosine receptors on dendritic cell surface enables T-cell costimulation through CD26 crosslinking, which enhances T-cell activation and proliferation. Despite a large body of work on the actions of the ecto-enzyme ADA on T-cell activation, questions arise on whether ADA can also modulate dendritic cell maturation. To this end we investigated the effects of ADA on human monocyte derived dendritic cell biology. Our results show that both the enzymatic and non-enzymatic activities of ADA are implicated in the enhancement of CD80, CD83, CD86, CD40 and CCR7 expression on immature dendritic cells from healthy and HIV-infected individuals. These ADA-mediated increases in CD83 and costimulatory molecule expression is concomitant to an enhanced IL-12, IL-6, TNF-α, CXCL8(IL-8), CCL3(MIP1-α), CCL4(MIP-1β) and CCL5(RANTES) cytokine/chemokine secretion both in healthy and HIV-infected individuals and to an altered apoptotic death in cells from HIV-infected individuals. Consistently, ADA-mediated actions on iDCs are able to enhance allogeneic CD4 and CD8-T-cell proliferation, globally yielding increased iDC immunogenicity. Taken together, these findings suggest that ADA would promote enhanced and correctly polarized T-cell responses in strategies targeting asymptomatic HIV-infected individuals.

## Introduction

Adenosine deaminase (ADA, EC 3.5.4.4) is a key enzyme in the purine pathway catalyzing the irreversible deamination of adenosine or 2′-deoxyadenosine to inosine or 2′-deoxyinosine and ammonia. ADA’s function is essential in maintaining an immune response as patients with ADA deficiency suffer from SCID, a rare inherited metabolic disorder that causes lymphopenia and immunodeficiency [1], [2] due to the accumulation of toxic substrates [3] and excessive adenosine receptor activation [4]. ADA is released into the extracellular medium by immune cells where the enzyme can bind to certain membrane proteins such as CD26 [5]–[7] and adenosine receptors A_1_ [8]_,_ A_2B_ [9] and A_2A_ [10]_._ CD26 is a T-cell-activation marker molecule that has dipeptidylpeptidase enzymatic activity. Physiologically, this activity regulates the actions of several peptides, including chemokines [11]. Adenosine receptors are members of the G protein coupled receptor family. A_1_ is negatively coupled to adenylate cyclase, while A_2A_ and A_2B_ can activate this same enzyme through the G_s_ protein subunit. The latter leads to an increase in cAMP levels, which may account for the adenosine mediated anti-inflammatory effects on T-cells, neutrophils or antigen presenting cells [12]. Thus, ADA is expressed as an ecto-enzyme with relevant physiological roles in the immune synapse [13], [14]. On one hand, the enzymatic activity of ADA reduces adenosine levels available for stimulation of adenosine receptors expressed on the T-cell surface contributing to immune regulation [7], [15], [16]. On the other hand, ADA delivers costimulatory signals to T-cells through CD26 crosslinking [17]. By acting as a bridge between A_2B_ adenosine receptors on dendritic cells (DCs) and CD26 on T-cells [13], ADA acts as a costimulatory molecule in DC-T-cell cocultures enhancing T-cell proliferation, Th-1/pro-inflammatory cytokine secretion [13], and T-CD4^+^ cell activation, memory, and Foxp3^+^ generation in both healthy and HIV-infected subjects [18]. Although HIV gp120 envelope protein disrupts the ADA-CD26 interaction [19], possibly contributing to the immunodeficiency [20], ADA is still able to enhance autologous T-cell proliferation against inactivated-HIV presentation by DC in individuals under HAART [21], suggesting a beneficial role for ADA on improving HIV-specific T-cell responses in those individuals [22].

While ADA actions on T-cells have been extensively studied, its actions on human DC are still largely unknown, despite the pivotal role of these cells on the immunological synapse and T-cell activation. In fact, DCs are the most potent antigen presenting cells (APC), critical in the initiation and control of protective immune responses [23], [24]. Immature dendritic cells (iDCs) are widely spread among peripheral tissues, where they actively scan the environment to detect any invading pathogen or foreign antigen. Towards this end, pathogen associated molecular patterns (PAMPS), nucleotides, inflammatory cytokines or cell damage activate a complex DC maturation program consisting of the up-regulation of maturation markers such as CD83, costimulatory (such as CD80, CD86 and CD40) and HLA molecules, the activation of CCR7 expression-induced migration to secondary lymph-nodes and the secretion of T-cell polarizing cytokines, in a process set to efficiently activate antigen-specific T-cells [25]. In contrast, in the absence of alarm signals, self-antigens are presented to T-cells in the absence of or in limited costimulation, which results in tolerance, either by T-cell apoptosis, anergy or regulatory T-cell (Treg) development in a process driven by inhibitory receptors such as PD-1 or CTLA-4 [26] and suppressive mediators such as IL-10, TGF-β or adenosine [27]–[29]. These unique and versatile properties of DCs makes them a valuable tool for therapeutic vaccination purposes such as in HIV [30], [31], cancer [32], [33] or tolerogenic approaches [34]. Therefore, it is important to know the role played by ADA on the biology of DCs.

Since it has been observed that DCs are able to arise from monocytes *in vivo* [35], [36], monocyte-derived DCs are a good model for *in vitro* studies [37]. In this study we have addressed the role of ADA on the expression of costimulatory molecules, on cytokine and chemokine secretion, on the immunogenicity, migration and viability of human monocyte-derived DC from healthy or HIV-infected donors. Four novel observations derive from this study: First, ADA enhances DCs costimulatory molecule expression. Second, ecto-ADA actions on iDCs are able to increase the secretion of both pro-inflammatory cytokines and chemokines that are known to promote Th-1 immune responses. Third, we establish that both the enzymatic and enzymatic-independent role of ADA participate in ADA-induced effects. Finally, ADA globally enhances the immunogenicity of human DCs, a process that results in improved CD4^+^ T-cell proliferation after alloantigen presentation. These results demonstrate that ADA can lead to an increased mature DC status; a property that can be harnessed to improve the outcome of immune responses to chronic infections such as HIV.

## Materials and Methods

### Sampling and Study Population

Blood samples were obtained by venipuncture from the antecubital vein using EDTA-treated vacutainers (Becton Dickinson, Mountain View, CA, USA) and were processed immediately after extraction. Healthy control volunteers (n = 25) and HIV-1-infected individuals (n = 22) were included in the study. The study received the approval of the Institutional Committee of Ethics and Clinical Investigation and all individuals gave informed written consent. The characteristics of HIV- infected subjects are shown in Table 1.

**Table 1:** Clinical information of patients with HIV-1.

Patients Receiving HAART (n = 11)															
Sex	Age	VL*	T CD4				T CD4 Nadir				T CD8				Progression
			cells/µL		%		cells/µL		%		cells/µL		%		
**M**	48	<DL	582		28		255		28		882		42		NC
**M**	68	<DL	565		24		277		14		1354		66		NC
**M**	33	<DL	1075		34		396		16		1458		45		VC
**M**	40	<DL	666		42		537		30		458		21		NC
**M**	63	<DL	539		23		266		20		1174		51		NC
**M**	40	<DL	671		22		243		19		1456		47		NC
**F**	51	<DL	342		11		332		10		2533		79		VC
**F**	59	<DL	1245		41		185		35		1435		48		NC
**F**	44	<DL	625		37		274		28		809		48		NC
**M**	35	2,7	764		45		365		26		673		40		NC
**M**	37	2,7	1193		31		783		29		2297		59		VC
**Mean**		2,7	751,5		30,7		355,7		23,2		1320,8		49,6		
**SD**			291,4		10,3		170,5		7,8		641,4		14,9		
**Patients Not Receiving HAART (n = 11)**															
**Sex**	**Age**	**VL** *		**T CD4**				**T CD4 Nadir**				**T CD8**			**Progression**
				**cells/µL**		**%**		**cells/µL**		**%**		**cells/µL**		**%**	
**M**	60	<DL		420		16		420		16		1182		45	VC
**M**	65	1,6		512		26		504		28		874		44	EC
**F**	54	1,9		540		20		451		19		854		52	EC
**M**	48	2,1		876		31		823		30		1355		48	VC
**M**	36	3,1		576		32		197		30		1332		59	VC
**M**	34	3,2		519		25		406		29		1148		55	NC
**M**	31	3,4		480		30		480		30		76		48	VC
**M**	35	3,4		660		22		452		16		1368		46	NC
**F**	47	3,4		844		47		702		44		638		35	EC
**M**	35	3,5		880		40		867		36		1582		48	N/A
**M**	41	3,5		664		30		537		17		979		44	NC
**Mean**		2,9		633,7		29,0		530,8		26,8		1035,3		47,6	
**SD**		0,8		165,7		8,9		195,7		9,0		421,2		6,3	

* VL is expressed as log viral copies/ml.

NC: Non controller.

VC: Viremic Controller.

EC: Elite Controller.

### Antibodies and Reagents

FITC-conjugated mAbs against HLA-DR, HLA-ABC, CD4, CD14, CD19, CD25, CD45RA and IgG-γ1 isotype-matched control, PE-conjugated mAbs against HLA-DR, CD1a, CD11c, CD14, CD19, CD25, CD40, CD45RO, CD56 and IgG-γ1 isotype-matched control, PerCP-conjugated mAbs against CD3, CD4 and IgG-γ1 isotype-matched control and PE-Cy7-conjugated mAb against CCR7 from Becton Dickinson Biosciences (Erembodegem-Aalst, Belgium); PE-conjugated mAbs against CD80, CD83 and CD86 from Coulter-Immunotech Diagnostic (Marseille, France); PE-conjugated mAb against CD209 from eBioscience (California, USA). Human-specific mAb against CD26, TA5.9-CC1-4C8 were used as previously indicated [13], [19], [20].

### ADA Preparation and Activity Determination

ADA from calf intestine (Roche Diagnostic Inc, Mannheim, Germany) was desalted by passage through a PD-10 column (Amersham Biosciences, Cerdanyola, Spain) and its enzymatic activity was evaluated by adenosine deamination measured as decreases in absorbance at 265 nm as previously reported [38]. To obtain ADA without enzymatic activity, desalted ADA was treated with HgCl_2_ as described previously [8] and passed again through a PD-10 column to eliminate remaining HgCl_2_. The enzymatic activity of Hg^2+^-treated ADA (ADA-Hg) was completely abolished. The possibility of LPS contamination in the ADA preparations was addressed by boiling the ADA preparation for 10 min before its addition to iDCs cultures. This process completely removed ADA’s ability to induce phenotypic changes in iDCs (data not shown).

### Generation of Monocyte-derived DCs

Monocyte-derived DCs were obtained as described previously [13], [18], [39]. Human PBMC were obtained immediately after blood extraction using the standard Ficoll gradient method. To obtain monocytes, PBMC were incubated for 2 h at 37°C in a humid atmosphere of 5% CO_2_ in 75 cm^2^ plastic flasks with DC-medium, consisting of serum free XVIVO-15 medium (Bio-Whittaker, Walkersville, MD, USA) supplemented with 1% autologous serum, 50 µg/ml gentamycin (Braun B., Melsungen, Germany), 2.5 µg/ml fungizone (Bristol-Myers Squibb, Munchen, Germany) and, in the case of HIV-infected individuals, with 1 µM zidovudine (Retrovir from GlaxoSmithKline, Madrid, Spain) to avoid possible replication of endogenous HIV-1. To obtain immature DCs (iDCs) adherent monocytes were washed four times with pre-warmed serum free XVIVO-10 medium (Bio-Whittaker) and cultured during 5 days in DC-medium, supplemented at days 0 and 2 with human recombinant IL-4 and GM-CSF (1000 UI/ml, each) (Prospec-Tany Technogene LTD, Rehovot, Israel). The purity and immunophenotype were assessed by flow cytometry and was found to be similar to previously reported [13], [18], [39]. At day 5 of differentiation, iDCs were washed 4 times with XVIVO-10 media, counted and 5·×10^5^ cells/ml distributed in 12.5 cm^2^ plastic flasks with fresh DC-medium supplemented with IL-4 and GM-CSF. To obtain ADA-treated DCs or fully mature DCs (mDCs), 2 µM ADA or 10 µl/ml of cytokine maturation cocktail were added, and cultured for two additional days. This cytokine maturation cocktail contains TNF-α, IL-6 (1000 UI/ml each, Strathmann-Biotec AG), IL-1β (300 UI/ml, Strathmann Biotec AG) and PGE_2_ (1 µg/ml, Pfizer, Madrid, Spain).

### T-cell Isolation, Cocultures and Proliferation Assays

As a source of T-cell-enriched population, non-adherent PBMC (see above) were washed four times with XVIVO-10 medium and collected for further coculture experiments as described before [13], [18]. To measure T-cell proliferation, 10^7^ cells/ml were stained with 5 µM CFSE using the CellTrace CFSE proliferation kit (Molecular Probes, Paisley,UK) as indicated by the manufacturer’s protocol.

Autologous or allogeneic cocultures were performed in 96-well round bottom plates, containing non-treated or treated iDCs (10^4^ cells/well) and fresh CFSE stained T-cells (2·×10^5^ cells) in a final volume of 200 µl of in XVIVO-10 media. T-cell proliferation was assessed by flow cytometry after 7 days of coculture in a humidified atmosphere with 5% CO_2_ at 37°C.

### Cytokine and Chemokine Secretion Measurement

iDCs, ADA-treated iDCs or mDCs (5·×10^5^ cell/ml) were cultured in DC-medium and the secretion of cytokines and chemokines was measured in the supernatants after 48 h of culture. Multiplex Luminex assays (Cytokine Human 25-Plex Panel, Invitrogen, Carlsbad, CA, USA) were performed following the manufacturer instructions. The following 25 mediators were tested: Eotaxin, GM-CSF, IL-1β, IL-1RA, IL-2, IL-2R, IL-4, IL-5, IL-6, IL-7, CXCL8(IL-8), IL-10, IL-12p40/p70, IL-13, IL-15, IL-17, IFN-α, IFN-γ, CXCL10(IP-10), CCL2(MCP-1), CXCL9(MIG), CCL3(MIP1-α), CCL4(MIP-1β), CCL5(RANTES) and TNF-α.

### Chemotaxis Assay

DCs migration towards CCL19 or CCL21 chemokines (from PeproTech, London, UK) was measured in a 96-plate transwell chemotaxis chambers (Corning Costar, Cambridge, MA, USA), using a polycarbonate filter of 5 µm pore size as previously reported [40]. Briefly, 150 µl of recombinant human CCL19 (300 ng/ml) or recombinant human CCL21 (250 ng/ml) or medium alone were placed in the lower wells. Upper wells were loaded with 50 µl of XVIVO-15 medium containing 2.5·×10^4^ iDCs, ADA-treated iDCs or mDCs. Each condition was set up in duplicates. The complete chamber was kept in a humidified atmosphere with 5% CO_2_ at 37°C for 3 h. Thereafter, cell suspensions in the upper well were removed and cells that had migrated through the filter to the lower wells were counted by flow cytometry for 1 min. Values were given as percentage of migrated cells ± SEM in relation to the initial cell input.

### Flow Cytometry

Cells were collected, washed with PBS and incubated 30 min at 4°C with PBS containing 10% rabbit serum and 0.1% NaN_3_ prior 30 min incubation (4°C) with primary labeled antibodies. When CCR7 staining was performed, cells were incubated with PBS containing 5% BSA for 30 min at room temperature prior staining with CCR7-labeled monoclonal antibody for 30 min at 4°C. Cells were washed with PBS and 10,000 to 50,000 events were collected on a FACsCAN or a FACsCANTO flow cytometer (Becton Dickinson Biosciences Erembodegem-Aalst, Belgium). Data was analyzed with FlowJo software (Tree Star, Inc. Ashland, OR, USA). The expression of iDCs immunophenotype (CD3^−^, CD14^−^, CD19^−^, CD56^−^, HLA-DR^+^, HLA-ABC^+^, CD80^low^, CD83^low^, CD86^+^, CD1a^+^, CD11c^+^, CD40^+^, CD45RO^+^, CD45RA^−^) was addressed and the percentage of positive cells and the geometric mean fluorescence intensity was measured. DC and T-cell populations were selected by forward and side light-scatter parameters.

### Cell Death Assay

iDCs, ADA-treated iDCs and mDCs were carefully washed with XVIVO-15 medium after 48 h cell culture in DC-medium. Cells (2·10^5^) were resuspended in 200 µl of XVIVO-15 media and incubated with 40 nM of the potentiometric mitochondrial probe DIOC_6_ (Molecular Probes; Invitrogen, Carlsbad, California, USA) and 10 µl of propidium iodide (Sigma-Aldrich, St. Louis, MO, USA) for 1 h at 37°C. Samples were then acquired in a FACsCANTO instrument (BD). A minimum of 10,000 events of every sample were collected and analyzed on FlowJo software. After gating on DCs population by Forward and Side parameters, apoptotic and necrotic cells were identified by their low DIOC_6_ fluorescence [41] plus the absence or presence of propidium Iodide staining.

### Statistical Analysis

Graphs were plotted using the GraphPad Prism 5.0 software (GraphPad Software, Inc., San Diego, California, USA). Quantitative variables were analyzed using medians and IQRs. The non-parametric Mann Whitney *U*-test for unpaired data or the Wilcoxon signed rank test for paired data were used when comparing two groups. For multiple comparisons, Kruskal-Wallis test followed by Dunns post-test was used. For all the tests used, a two tailed *P* value <0.05 was considered statistically significant.

## Results

**Figure 1:**
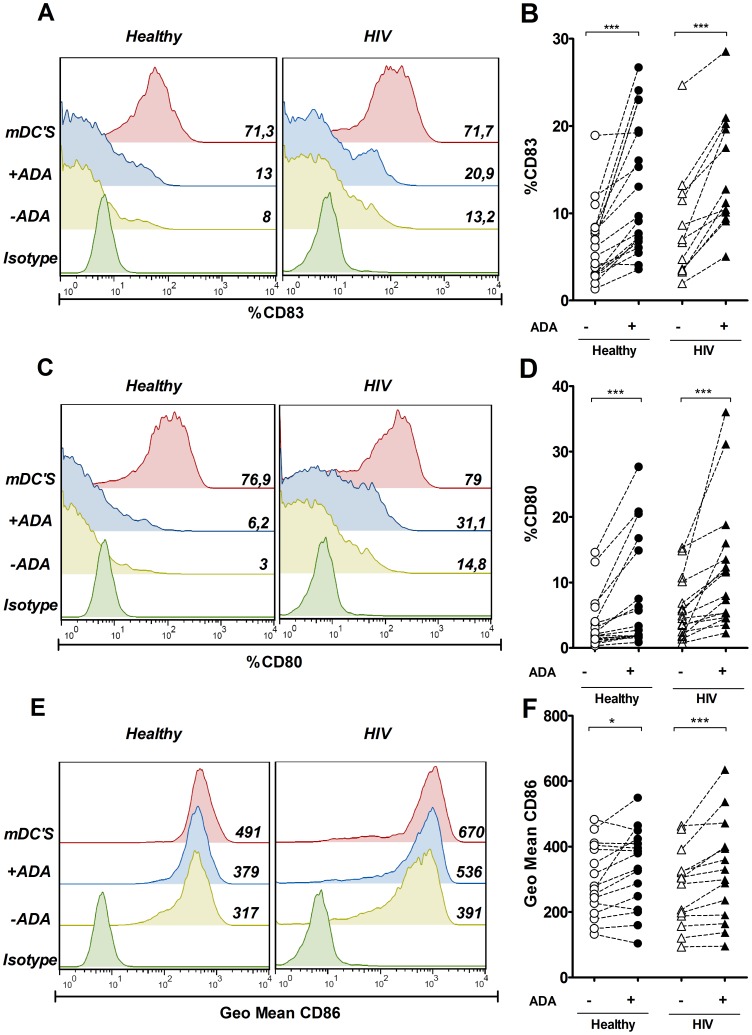
ADA enhances CD83, CD80 and CD86 expression on iDCs. iDCs, obtained as indicated in the Materials and Methods, were cultured for 48 h in medium in the absence (−ADA) or in the presence (+ADA) of 2 µM ADA or in the presence of maturating cocktail (mDCs). Expression of CD83 (A and B), CD80 (C and D) or CD86 (E and F) in the DCs gate was assessed by flow cytometry. In A, C and E, histogram overlays and the percentage of positive cells (A and B) or geometric mean (C) for a representative healthy donor and HIV-infected subject are shown. In B, D and F, values obtained from 16 to 19 healthy donors (circles) or 12 to 16 HIV-infected individuals (triangles) in the absence (open symbols) or in the presence (filled symbols) of 2 µM ADA are plotted. Each pair of linked symbols represents results from a particular individual. *P<0.05; ***P<0.001.

**Figure 2:**
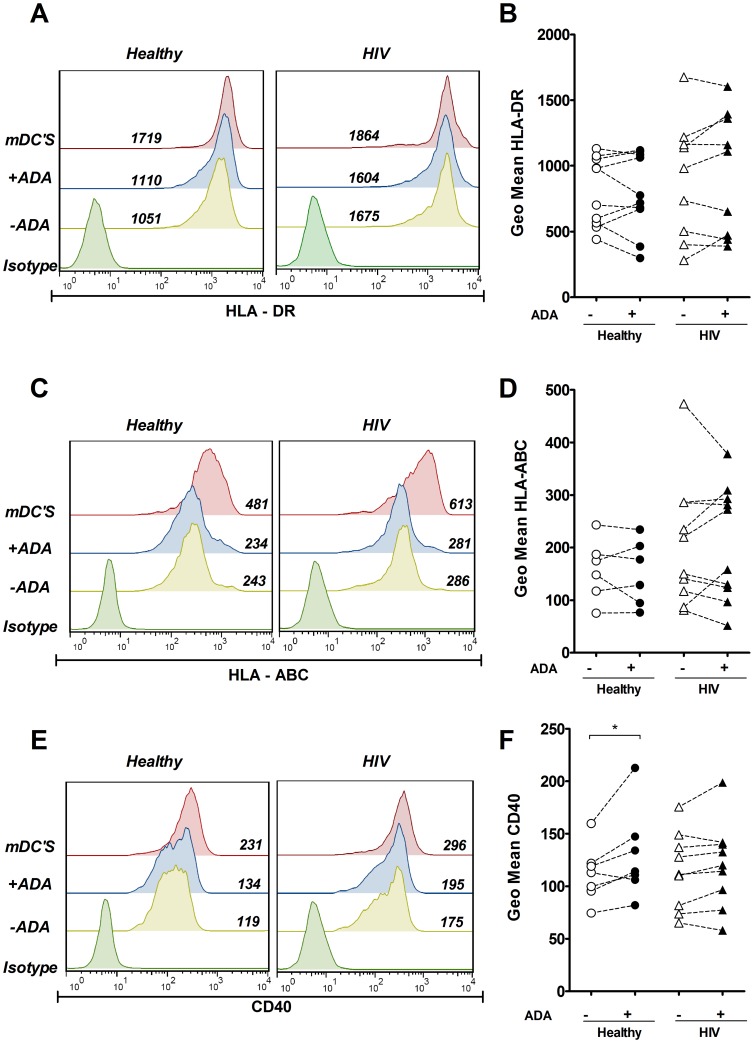
ADA effect on CD40 and HLA expression on iDCs. iDCs, obtained as indicated in the Materials and Methods, were cultured for 48 h in medium in the absence (−ADA) or in the presence (+ADA) of 2 µM ADA or in the presence of maturating cocktail (mDCs). Expression of HLA-DR (A and B), HLA-ABC (C and D) or CD40 (E and F) in the DCs gate was measured by flow cytometry. In A, C and E, histogram overlays and the geometric mean from a representative healthy donor and HIV-infected patient are shown. In B, D and F, values obtained from 6 to 10 healthy donors (circles) or 9 to 10 HIV-infected subjects (triangles) in the absence (open symbols) or in the presence (filled symbols) of 2 µM ADA. Values obtained from 6 to 10 healthy donors (circles) or 9 to 10 HIV-infected subjects (triangles). * *P*<0.05.

ADA enhances the expression of costimulatory molecules in iDCsTo study the influence of ADA on the phenotype of DCs, we first investigated the effect of ADA on the expression of costimulatory molecules by flow cytometry. Immature monocyte-derived DCs (iDCs) from healthy or HIV-infected donors were cultured in the absence or in the presence of ADA for 48 h, and the expression of CD83, CD80 and CD86 was compared with the expression detected in mature DCs (mDCs) obtained from the same donors after a cytokine-PGE_2_ maturation cocktail stimulation [42]. Different doses of ADA were tested, choosing 2 µM as the lowest dose at which significant ADA effects were observed (Fig. S1 A and B). When iDCs were treated with ADA, a consistent and statistically significant (*P*<0.001) upregulation of CD83 (Fig. 1 A and B) and CD80 (Fig. 1 C and D) was observed, both in healthy and in HIV-infected donors. As expected, mDCs showed a marked increase in the expression of CD83 and CD80 (Fig. 1 A and C). When assessing CD86 expression (Fig. 1 E and F), all DCs expressed CD86 (>95% cells were positive for this marker) but culturing iDCs in the presence of ADA resulted in a statistically significant increase in the CD86 geometric mean, which was seen in healthy donors (*P*<0.05) as well as in HIV-infected individuals (*P*<0.001) (Fig. 1 F). Again, mDCs expressed a higher CD86 geometric mean than its immature counterparts. CD83, CD80 and CD86 are classically considered maturation markers [43], and their upregulation suggests that ADA is potentiating DC maturation. The coordinated upregulation of costimulatory and HLA molecules on the surface of DCs is a characteristic feature of maturing DCs [43], thus, we next sought to examine the ADA effect on HLA expression in the same experimental settings as above. No statistically significant changes in the geometric mean of HLA-DR (Fig. 2 A and B) or HLA-ABC (Fig. 2 C and D) were obtained in either healthy nor in HIV-infected individuals when their iDCs were cultured in the presence of ADA. In contrast, cytokine maturation of DCs clearly increased the HLA-DR and HLA-ABC geometric means (Fig. 2 A and C). Both ADA treatment and cytokine maturation cocktail failed to affect the percentage of cells expressing other monocyte-derived DCs markers such as CD11c (90% positive) or CD14 (<5% positive). The failure of ADA to induce a clear upregulation of HLA molecules indicates that ADA does not promote a full maturation process but a preferential enhancement of costimulatory molecules. To address the latter, the ADA effect on the expression of CD40, another costimulatory marker, was measured. All iDCs expressed CD40 (>95% cells were positive on this marker). A moderate and significant (p<0.05) increase in CD40 geometric mean was observed when iDCs from healthy donors were treated with ADA (Fig. 2 E and F). A tiny increase in CD40 geometric mean was noticeable when iDCs from HIV-infected individuals were treated with ADA, although this effect was not consistent on every patient tested due to a high inter-individual variability (Fig. 2 F). In contrast, a clear increase in geometric mean was observed both in healthy and HIV individuals in mDCs (Fig. 2 E). Considering that CD83, CD80, CD86 and CD40 are important for delivering the appropriate secondary signals to T-cells [43], [44] the ADA-mediated up-regulation of these markers indicates that ADA may increase the costimulatory potential of iDCs. Given the heterogeneity of the different HIV-infected individuals (table 1) we sought to determine whether differences could be observed on iDCs by classifying patients according to their clinical parameters, including CD4^+^ or CD8^+^ cell counts, viral load, HAART treatment or disease progression (controller vs non controller). This analysis shows that iDCs from patients with detectable viremia have increased CD80 while reduced CD83 expression compared to patients with undetectable viremia (Fig. S2 B). No other major differences were found on this analysis. We further tested whether the ADA effects on costimulatory markers expression might correlate with any clinical parameter from these patients. Interestingly, ADA-mediated effects on CD80 expression did correlate with the patients’ viral load (Fig. S3 A), but no other major correlations were found.

### ADA Enhances Th-1/pro-inflammatory Cytokine and Chemokine Secretion

**Figure 3:**
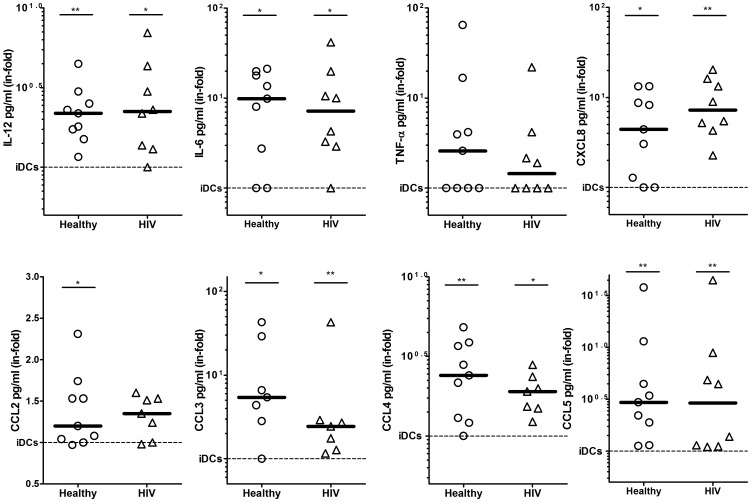
ADA increases cytokines and chemokines secretion. iDCs, obtained as indicated in the Materials and Methods, from 9 healthy (circles) and 8 HIV-infected (triangles) donors were cultured during 48 h in medium in the absence (iDCs) or in the presence of 2 µM ADA and the indicated cytokines and chemokines were determined in the supernatant as described in the Materials and Methods. Values are expressed as the ratio (in-fold) of cytokine or chemokine levels obtained in the presence of ADA versus levels obtained in the absence of ADA (iDCs, the reference value of 1 is represented by a dotted line). For each group, the median is indicated by a thick line. *P<0.05; **P<0.01 with respect to iDCs.

Eliciting the appropriate cytokine and chemokine expression pattern to each invading pathogen is critical to ensure a correctly polarized and efficient immune response [25]. To investigate whether ADA was able to enhance DC cytokine and chemokine release and to characterize the specific molecules involved, we analyzed the expression of 25 cytokines and chemokines in culture supernatants from non-treated and ADA-treated iDCs. iDCs from either healthy or HIV-infected individuals were cultured in the absence or in the presence of ADA or with the maturation cocktail. After 48 h supernatants were collected for further analysis. ADA addition to iDCs cultures resulted in a consistent enhancement in the release of a specific group of Th-1/pro-inflammatory cytokines and chemokines both in healthy and in HIV donors (Fig. 3). IL-12, whose secretion is pivotal in regulating Th-1 polarization, was augmented 2-fold in the presence of ADA when compared to iDCs in both healthy and HIV-infected individuals. When the pro-inflammatory cytokines were addressed, a striking 10-fold up-regulation of IL-6 was found, whereas a less consistent increase in TNF-α (2.5 median in-fold) was seen (Fig. 3). It must be noted that IL-1β which is often found together with IL-6 and TNF-α under inflammatory stimuli, was not detected in our iDC cultures in any donor, suggesting that a very specific effect was triggered. Low, yet measurable levels (40–50 pg/ml) of the antiviral IFN-α cytokine were detected in iDCs, but were not increased by ADA (Fig. S1 C). When chemoattractant mediators were analyzed in the supernatants, a clear and significant increase in CXCL8, CCL3, CCL4 and CCL5 was observed in healthy donors and also in HIV-infected individuals (Fig. 3). The ADA-mediated increase on these latter three CCL chemokines (β-chemokines) is relevant to HIV pathogenesis since potent HIV-inhibitory activities are associated to these mediators [45]. In addition, ADA induced a less prominent but noticeable increase in other chemokines such as CCL2 (Fig. 3) or CXCL9 (1.35 median in-fold, Fig. S1). Importantly, the cytokine and chemokine profile enhanced by ADA is very similar to the one observed for mDCs (Fig. S1). It is also remarkable that in our culture conditions no measurable levels of eotaxin, IL-5, IL-7, IL-13, IL-15, IL-17 and CXCL10 were detected (data not shown).

### ADA Enzymatic–dependent and Independent Activities Contribute to its Effects on iDCs

**Figure 4:**
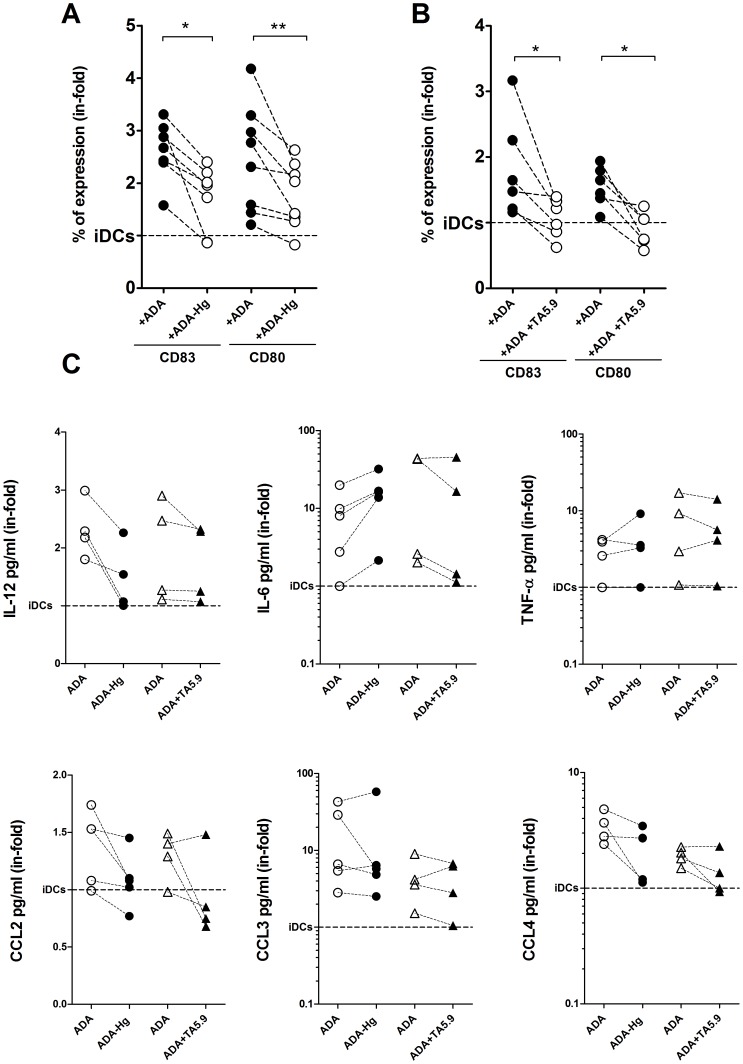
Enzymatic and non-enzymatic activities are implicated on ADA-mediated effects. iDCs, obtained as indicated in the Materials and Methods, from 4 to 8 healthy donors were cultured for 48 h in medium in the absence (iDC) or in the presence of 2 µM ADA (+ADA) or 2 µM HgCl_2_ inactivated ADA (ADA-Hg) or iDCs were pre-incubated with the mAb anti-CD26 TA5.9 and incubated with 2 µM ADA (ADA+TA5.9). In (A) and (B), the expression of CD83 and CD80 was assessed in the DCs gate by flow cytometry. In C) the indicated cytokines and chemokines were determined in the supernatants after 48 h of cell culture. Each pair of linked symbols represents results from a particular individual. Results are expressed as the ratio (in-fold) of the values obtained in the presence of ADA, ADA-Hg or ADA+TA5.9 versus untreated cells (iDCs, the reference value of 1 is represented by a dotted line). *P<0.05; **P<0.01 with respect to iDCs.

Both adenosine deamination and CD26 binding are reported to have important roles on the immune system [7], [14]. Thus, we sought to delineate the particular contribution of these activities on the ADA-mediated effects on iDCs. To address this question, we blocked the enzymatic activity of ADA via inhibition with HgCl_2_ (ADA-Hg). ADA-Hg is unable to degrade adenosine while retaining its receptor binding capability [8]. iDCs were then cultured in the absence or presence of ADA or ADA-Hg. As expected, ADA led to an upregulation of CD83 and CD80 expression on all donors tested (Fig. 4 A and B). By contrast, ADA-Hg decreased the ADA-mediated up-regulation of CD83 and CD80 in a median of 0.7 and 0.8 in-fold respectively (Fig. 4 A). Despite this reduction, ADA-Hg was still able to induce a noticeable increase in CD80 and CD83 expression compared to non-treated iDCs (Fig. 4 A), indicating that a mechanism other than the enzymatic activity must also be involved. Since the presence of ADA on cell membranes has classically been associated to CD26 expression in many cell types [5], [46], [47] and CD26 is also expressed in human iDCs [13], the participation of CD26 on ADA-mediated effects on iDCs was investigated. To address this, the mAb TA5.9, which is directed against the ADA-binding epitope on CD26, was used. When iDCs had been previously pre-incubated with TA5.9, the ADA-mediated upregulation of CD83 and CD80 was reduced by 0.46 and 0.64 in-folds respectively suggesting an important role for ADA binding to CD26 in the ADA-mediated increase in CD86 and CD80 expression (Fig. 4 B). Our next goal was to address the role of ADA enzymatic activity and CD26 binding on cytokine and chemokine release. iDCs cultured in the presence of ADA-Hg have a decreased secretion of IL-12, CCL2, CCL4 and slightly CCL3 (Fig. 4 C) while TNF-α secretion was not significantly modified and IL-6 secretion was enhanced under these conditions (Fig. 4 C). When the mAb TA5.9 was used in the same experimental settings, no significant effect could be seen on IL-12, TNF-α or CCL3 secretion, while IL-6, CCL2 and CCL4 were slightly decreased (Fig. 4 C). Interestingly, neither the ADA-mediated changes in CXCL8 and CCL5 secretion were affected by ADA-Hg or TA5.9 (results not shown), suggesting these require a mechanism independent of CD26 or adenosine levels. Taken together, these results suggest that both the enzymatic activity and binding to CD26 contribute to the global ADA mediated effects observed, since the abrogation of one of these mechanisms alone does not abolish all the ADA effects.

### ADA Enhances the DCs Capacity to Stimulate Allogeneic T-cells

**Figure 5:**
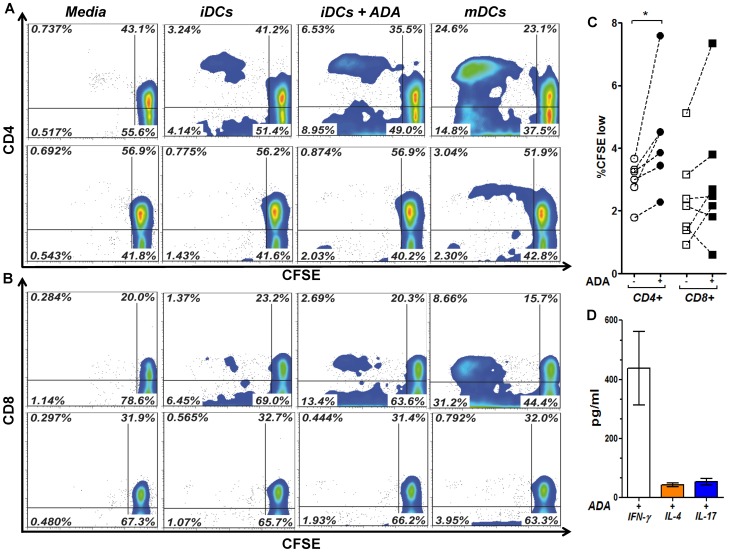
ADA enhances DCs immunogenicity in iDC-T-cell Allogeneic cocultures. In A and B, iDCs, obtained as described in the Materials and Methods, from a representative healthy donor were cultured during 48 h in medium in the absence of ADA (iDCs), in the presence of 2 µM ADA (iDCs+ADA) or in the presence of maturating cocktail (mDCs). DCs were washed and cocultured with allogeneic (upper contour plots) or autologous (lower contour plots) T-cells (1∶20 DCs:T-cells ratio). After 7 days, the percentage of CD4^+^ (A) and CD8^+^ (B) T-cell proliferation was assessed by flow cytometry using the CFSE method. In (C) percentages of CD4^+^ (circles) and CD8^+^ (squares) T-cell proliferation in the absence (open symbols) or the presence (filled symbols) of 2 µM ADA in allogeneic cocultures from 6 to 7 healthy donors are shown. Each pair of linked symbols represents results from a particular healthy donor. *P<0.05. In (D) bars indicate IFN-γ, IL-4 and IL-17 levels in ADA-treated iDC co-culture supernatants. Results are the mean ± SD (pg/ml) of 3 independent experiments.

The ADA-induced enhancement of costimulatory molecules expression and Th-1/pro-inflammatory mediators release on iDCs, suggested that ADA could render iDCs more immunogenic by improving antigen presentation. To address this question, iDCs were cultured in the absence or presence of ADA or with maturating cocktail during 48 h, strictly washed, and cocultured with allogeneic CFSE-stained T-cells or autologous CFSE-stained T-cells as a negative control. Proliferation of both CD4^+^ and CD8^+^ T-cell subsets was quantified. As expected, mDCs induced the highest T-cell proliferation (Fig. 5 A and B). Remarkably, ADA also induced a significant increase in CD4^+^ T-cell proliferation compared to non-treated iDCs (Fig. 5 A and C). Moreover, an enhanced proliferation of CD8^+^ T-cells was also observed in cocultures with ADA-treated iDCs, although this increase was less consistent than that of CD4^+^ T-cells (Fig. 5 B and C). Barely detectable levels of CD4^+^ or CD8^+^ T-cell proliferation was detected in autologous cocultures, neither in the absence nor in the presence of ADA (Fig. 5 A and B), discarding the possibility of ADA being presented as a foreign antigen. As ADA has been previously shown capable of increasing Th-1 immune responses when present at the immune synapse [13], we further tested whether ADA-induced effects on iDCs could still favor this T-cell polarization. To address this issue, co-culture supernatants were addressed for IFN-γ, IL-4, and IL-17, indicative of Th-1, Th-2 and Th-17 polarization, respectively. In all conditions tested IFN-γ levels clearly predominated, and ADA actions on iDC did not alter this secretion profile, as we detected 423 pg/ml of IFN-γ as a median, while only 40 pg/ml of IL-4 and 49 pg/ml of IL-17 were found (Fig. 5 D). These results indicate that the ADA-induced effects on iDCs contribute to an improved iDC function.

### ADA Increases CCR7 Expression without an Increase in DCs Migration

**Figure 6:**
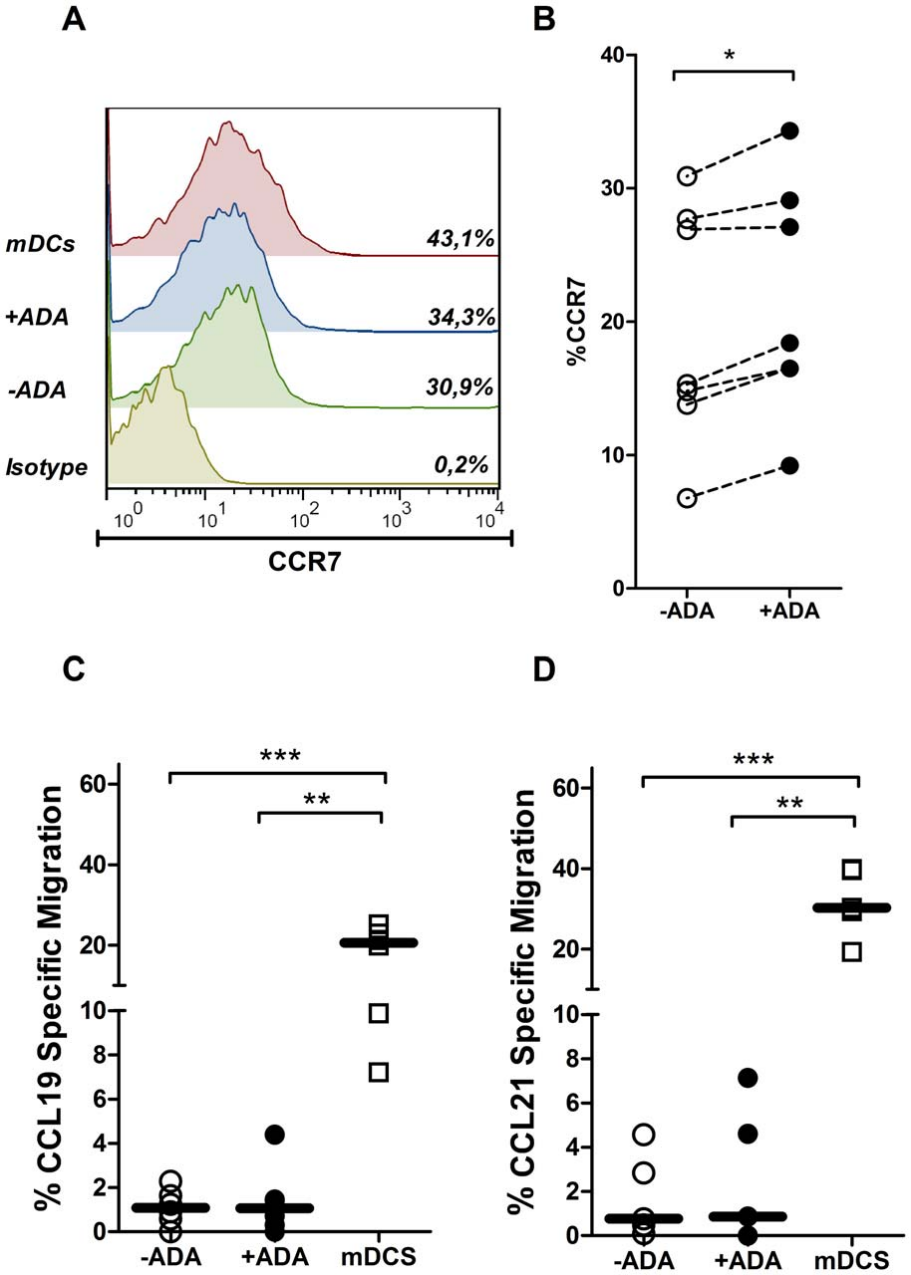
Effect of ADA on CCR7 expression and iDC migration. iDCs, obtained as described in the Materials and Methods, from healthy donors were cultured during 48 h in medium in the absence (−ADA) or in the presence (+ADA) of 2 µM ADA or in the presence of maturating cocktail (mDCs). In (A) and (B), CCR7 expression in the CD40^+^ DCs gate was measured by flow cytometry. Histogram overlays and the percentage of CCR7 expression for a representative donor are shown in (A). Values obtained from 7 healthy donors in the absence (open symbols) or in the presence (filled symbols) of 2 µM ADA are shown in (B). In (C) and (D), CCL19/CCL21 chemokine-induced migration assays were performed as described in the Materials and Methods. The percentage of cell migration to CCL19 (C) or CCL21 (D) in the absence (−ADA) or in the presence (+ADA) of 2 µM ADA or in the presence of maturating cocktail (mDCs), in relation to the initial cell input is shown. *P<0.01, ***P<0.001.

To further characterize the ADA effect on DCs function, we next tested the effect of ADA on DCs migration. CCR7 is critical to normal DC function as it drives antigen-loaded DCs entry to secondary-lymph nodes, through CCL19 and CCL21-dependent chemotactic gradients [48]. CCR7 is up-regulated upon DCs maturation, thus its expression is an indicator of DC status [43]. Therefore, we determined CCR7 expression and CCR7-dependent DCs migration as a way to evaluate the DC population. iDCs from healthy donors were cultured in the absence or in the presence of exogenous ADA or in the presence of maturating cocktail and CCR7 expression was determined by flow cytometry. A small but statistically significant increase in CCR7 expression was obtained in the presence of ADA in every tested healthy donor (Fig. 6 A and B). In accordance with previous studies [49], the maturation cocktail induced a more potent up-regulation of CCR7 (Fig. 6 A). We next sought to determine whether the increase on CCR7 expression also improved CCR7-dependent DC migration. To address this question, a transwell assay was used to assess the migration towards CCL19 or CCL21. As expected, extremely low numbers of iDCs migrated to CCL19 or to CCL21 after 3 hours of assay (Fig. 6 C and D). ADA addition to iDC cultures did not result in significant changes in iDC migration, neither to CCL19 nor to CCL21 in contrast to the migration observed with mDCs (Fig. 6 C and D). The ADA-induced increase in CCR7 expression fits with the role of ADA on increasing the expression of costimulatory molecules in iDCs that confers a more mature DC status, but is not sufficient to induce an increase on the in vitro DCs migration.

### Role of ADA in iDCs Viability

**Figure 7:**
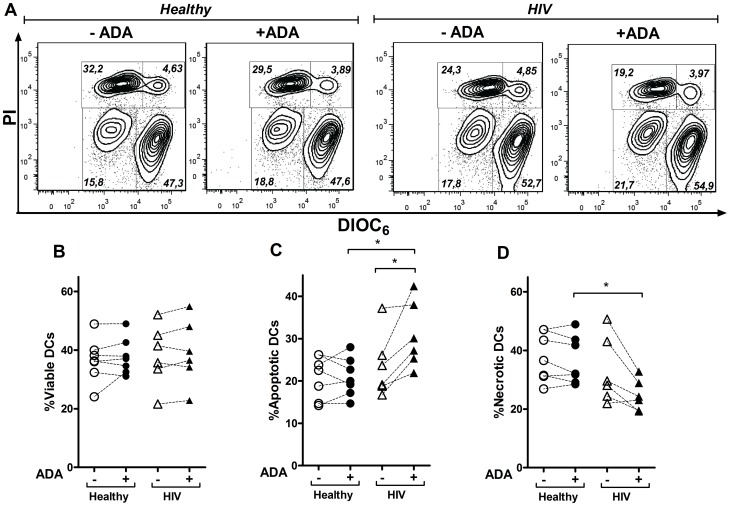
Effect of ADA on the iDCs viability. iDCs, obtained as described in the Materials and Methods, from healthy or HIV-infected donors were cultured during 48 h in the absence (−ADA) or in the presence (+ADA) of 2 µM ADA. Cell viability was assessed through DIOC_6_ and propidium iodide (PI) staining and measured by flow cytometry. In (A), contour plots showing the percentage of viable (bright DIOC_6_ and negative propidium iodide staining), apoptotic (low DIOC_6_ and negative propidium iodide staining) and necrotic (low DIOC_6_ and positive propidium iodide staining) populations from a representative healthy or HIV-infected donor are shown. Percentage of viable (B), apoptotic (C) and necrotic (D) DCs from 7 different healthy and 6 HIV-infected donors are shown.*P<0.05.

The right balance between cell death and survival is critical to achieve a correct immune homeostasis and this is especially important in HIV-infected donors where DCs death contributes to the pathogenesis of the disease [50]. Thus, we next tested whether the ADA-induced more mature DC phenotype could be playing a role on DCs viability. iDCs from either healthy or HIV-infected individuals were cultured in the absence or in the presence of ADA or with maturing cocktail. DIOC_6_, a fluorescent dye sensing mitochondrial potential was used to stain viable cells. Early apoptotic and late apoptotic/necrotic cells could be easily distinguished through the combination of low DIOC_6_ staining and negative (apoptosis) or positive (necrosis) propidium iodide staining (Fig. 7). The low percentage of cells that showed a bright DIOC_6_ and positive propidium iodide staining might represent dead cells phagocytosed by live DCs or DCs broken during manipulation. ADA addition to iDCs from healthy or HIV-infected donors did not result in significant changes in iDCs viability after 48 h of cell culture as measured by low DIOC_6_ staining (Fig. 7 A and B). When apoptosis and necrosis were addressed on iDCs from healthy donors, ADA was found unable of inducing significant changes in any of these populations, (Fig. 7 A, C and D). In contrast, all HIV-infected donors showed an increased percentage of early apoptotic cells in the presence of ADA while a reduced necrosis was detected (Fig. 7 A, C and D). The viability of DCs was clearly higher while reduced apoptosis and necrosis were observed in mDCs from either healthy or HIV-infected individuals (Fig. S4), a result most likely due to the presence of PGE_2_ in the maturating cocktail [51]. These findings indicate that ADA does not play a role in iDCs viability in healthy individuals whereas a different situation emerges on HIV-infected donors. In a chronic disease setting, ADA showed a protective role against late steps of cell death, an observation which may apply to other cells such as CD8 T-cells [52].This was however, not always reflected in a total increase in iDCs viability, suggesting that ADA is inhibiting a post-mitochondrial event in apoptotic cascades in iDCs from HIV-infected individuals.

## Discussion

In this study, we explored the effects of extracellular ADA on human monocyte-derived DCs from healthy and HIV-infected individuals. Four novel observations derive from this study. First, ADA added to human iDCs cultures enhances DCs-self costimulatory and maturation molecule expression. Second, we demonstrate, for the first time, that ecto-ADA actions on iDCs are able to increase the secretion of both pro-inflammatory cytokines and chemokines that are known to promote Th-1 immune responses. Third, we establish that both the enzymatic and enzymatic-independent role of ADA participate in ADA-induced effects. Finally, ADA globally enhances the immunogenicity of human DCs, a process that results in an improved CD4^+^ and CD8^+^ T-cell activation after alloantigen presentation.

Despite the now clear role of ecto-ADA on promoting an enhanced T-cell activation, proliferation and Th-1 effector/memory generation [13], [18], [21], it remained unclear whether ecto-ADA would modulate human DC functions. It is this issue we have investigated. Using human monocyte-derived DCs obtained from peripheral blood, we first determined if the addition of ADA to iDC cultures would induce the expression of costimulatory molecules on iDCs. In fact we observed that ADA increases CD83, CD80, CD86 and CD40 expression. These molecules are classically considered maturation markers and their up-regulation suggests that ADA is potentiating the DCs maturation. Considering that CD83, CD80, CD86 and CD40 are important for delivering the appropriate secondary signals to T-cells [43], [44], the ADA-mediated up-regulation of these markers indicates that ADA may increase the costimulatory potential of DCs. Another interesting aspect to highlight is that the addition of ADA to iDC cultures increases Th-1/pro-inflammatory cytokine release including IL-12, TNF-α and IL-6. This cytokine pattern is very similar to the ADA-increased effect on DC-T-cell autologous cocultures [13], indicating that ADA can act not only on lymphocytes but also on DCs to increase Th-1/pro-inflammatory cytokine release. Whereas IL-12 is critical to polarize T-cells towards Th-1, both IL-6 and TNF-α promote inflammation and the activation of both innate and adaptive immune responses including T-CD4^+^ memory generation [18], [53] and the maturation of DCs [54]. In addition, pro-inflammatory cytokines in general and IL-6 in particular have been shown to block Treg mediated suppression of DCs [55] as well as global Treg suppression capacity [56]. Together with deaminase activity, the ADA-induced secretion of pro-inflammatory cytokines would provide a local “suppression free” environment to facilitate T-cell activation. In addition to the cytokines, we also demonstrate, for the first time, that ADA addition to iDCs culture is able to increase the secretion of chemokines such as CXCL8, CCL2, CCL3, CCL4 and CCL5. The global role of these chemokines is to recruit immune cells such as neutrophils, monocytes, DCs, and activated T and B cells to the sites where antigen processing is taking place, in order to further develop the corresponding immune response [57].

To discern the exact mechanisms by which ADA is acting to induce DCs maturation is difficult but our results indicate that both the enzymatic and extraenzymatic activity of ADA are mediating its effects. By using ADA inhibited by HgCl_2_ we observed a reduced expression of costimulatory molecules and a decreased secretion of IL-12, CCL2, CCL3 and slightly CCL4 compared to active ADA, suggesting that the enzymatic activity of ADA is mediating part of these effects. ADA catalyzes the irreversible deamination of adenosine to inosine and ammonia decreasing the amount of extracellular adenosine able to bind to its receptors. It is known that human monocyte-derived DCs as well as plasmacytoid DCs express adenosine receptors [13], [58], [59] and adenosine, by binding to its receptors, modulates DC function, generally reducing the maturation and immunogenicity of stimulated DCs by enhancing IL-10 while diminishing IL-12, IL-6 and TNF-α secretion, lowering allo-stimulatory potential and limiting Th1 polarization [58]–[60]. Since the ADA-induced effects are the increase, not the decrease, of the expression or secretion of these molecules, part of the effects observed here can be due to the ADA-mediated reduction of adenosine available to bind adenosine receptors. This has been seen in mice where adenosine deamination by ecto-ADA on DCs surface was revealed to be critical to ensure DCs activation to TLR ligands [61]. Interestingly however, inactive ADA (ADA-Hg) is able to increase IL-6 secretion, rather than decreasing it. Since adenosine has been proposed to signal through A_2B_ receptors and promote IL-6 secretion on iDCs [62], [63] and ADA, by binding to A_2B_ increases the affinity and signaling of its ligands [9], it is plausible that ADA inactivation results in increased adenosine effects through its receptors. We observed that part of the ADA-mediated effects are independent of its enzymatic activity. In this respect, human or bovine ADA binds to human CD26 [5], [64], [65], whereas murine ADA neither binds to mouse nor human CD26 [66], suggesting relevant differences between human and mice models. We have previously described that ADA, by a mechanism independent of its enzymatic activity, binds to CD26 on T-cells enhancing cytokine release and T-cell costimulation [13], [18], [20]. To address the role of CD26 in the ADA-induced effects, the mAb TA5.9, which is directed against the ADA-binding epitope on CD26, was used. In the presence of the antibody, the ADA-mediated upregulation of CD83 and CD80 was reduced and the secretion of IL-6, CCL2 and CCL4 were slightly decreased suggesting a role for ADA binding to CD26 in these effects. Membrane-bound CD26 is known to interact with CD45 [67], a tyrosine phosphatase also present in dendritic cells. Since CD45 has been shown to modulate costimulatory molecule expression and TLR-induced cytokine secretion on murine DCs [68]–[70] it is plausible that after ADA binding, CD26 may interact and affect the subsequent Src kinases under CD45.

While ecto-ADA may attract immune effector cells through chemokine up-regulation, it clearly does not seem to promote iDCs migration to CCL19 or CCL21 gradients, despite the upregulated CCR7 receptor expression observed in presence of ADA. This would fit with a role of ADA in the facilitation of DCs maturation rather than inducing the entire process alone. Regardless, the above mentioned ADA-mediated effects converge into one ultimate purpose: render DCs more immunogenic. In fact, in cocultures of lymphocytes and iDCs previously treated with ADA it was observed an enhanced proliferation of CD4^+^ T-cells. As DCs were extensively washed to eliminate the ADA added before the coculture, this improved immunogenicity is likely attributable to the enhanced expression of costimulatory molecules previously demonstrated. It is also plausible that iDCs treated with ADA are able to release greater amounts of cytokines and chemokines upon T-cell contact, due to their more mature status.

Finally, we have examined the effects of ADA on dendritic cells from asymptomatic HIV subjects. HIV-1 viral particles are known for affecting several immune mechanisms in their compulsory search for immune evasion. DC costimulatory molecule expression [71] and even CD26/ADA interactions [19], [20] are found to be disrupted by HIV-1. In addition, HIV-1 deviates the appropriate Th-1 cell polarization towards Th-2 [72] and takes advantage of immunoregulatory mechanisms such as Tregs [73] or adenosine cell generation [74] to ensure the disease progression. Last but not least, diverse HIV factors are known to render DCs more sensitive to apoptosis [50]. In the asymptomatic HIV subjects addressed here, where limited or null viremia is found, ADA was able to enhance costimulatory molecule expression and Th-1 promoting cytokine/chemokine secretion, slightly enhancing DCs viability in some individuals. Taken together, these findings suggest that HIV-T-cell specific responses could be improved in the presence of ADA. In fact, previous experiments revealed that inactivated HIV-loaded DCs cocultured with autologous T-cells in the presence of ADA enhanced HIV-specific T-cell responses [21]. In addition, CCR5-binding chemokines CCL3, CCL4 and CCL5 are among the most potent natural HIV-1 suppression factors [45] known to block HIV cell fusion by antagonizing CCR5, an HIV co-receptor [75]. Also CXCL8 showed some transcriptional inhibition of HIV-1 in peripheral blood lymphocytes [76]. These facts raise the question of whether ecto-ADA mediated effects on iDCs could be accompanied of certain HIV-1-R5 suppressing activity, a hypothesis deserving future investigation. Taken together, the findings here described demonstrate that ADA enhances maturation and costimulatory molecule expression on iDCs from healthy and HIV-infected individuals, concomitantly to an enhanced IL-12, IL-6, TNF-α, CXCL8, CCL3, CCL4 and CCL5 cytokine/chemokine secretion and an altered transition to late apoptosis in HIV-infected individuals. As a whole these effects render DCs more immunogenic and suggest that ADA might promote enhanced and correctly polarized HIV-specific T-cell responses targeting asymptomatic HIV-infected individuals.

## Supporting Information

Figure S1 ADA dose-response in CD80/83 expression and cytokine secretion. iDCs, obtained as indicated in the Materials and Methods, were cultured for 48 h in medium in the absence (iDC) or in the presence of 1, 2, 4 or 6 µM ADA (+ADA) and the expression of CD83 (A) or CD80 (B) was addressed by flow cytometry in the DCs gate. Bars indicating the mean ± SEM of 2 independent experiments are shown. In (C) the indicated cytokines and chemokines were determined in the supernatant from iDCs cultured in the absence (iDCs) or in the presence of 1, 2, 4 µM ADA or in the presence of the maturating cocktail (mDCs). Bars indicating the mean and SEM of 4 different experiments are shown. Values are expressed as the ratio (in-fold) of CD83, CD80 or cytokine/chemokine levels obtained in the presence of ADA or mDCs versus levels obtained in the absence of ADA (iDCs, the reference value of 1 is represented by a dotted line).
(TIF)

Figure S2 CD80 and CD83 expression on iDCs from HIV-infected subjects according to different clinical parameters. iDCs from HIV-infected subjects were cultured for 48 h in medium in the absence (−ADA) or in the presence of 2 µM ADA (+ADA) and the expression of CD83 and CD80 was addressed by flow cytometry. Patients were separated according to receiving HAART or not (A), having undetectable (<DL) or detectable viral load (>DL) (B), being above or below 600 CD4^+^/µL (C) or above or below 900 CD8^+^/µL (D).*P<0.05, **P<0.01.
(TIF)

Figure S3 Correlation of ADA effect on CD83, CD80 and CD86 expression on iDCs from HIV subjects with different clinical parameters. iDCs from HIV-infected subjects were cultured for 48 h in medium in the absence (−ADA) or in the presence of 2 µM ADA (+ADA) and the expression of CD83, CD80 and CD86 was addressed by flow cytometry. The % of ADA increase on the expression of each marker was obtained by subtracting the percentage of expression in the absence of ADA from the the percentage of expression in the presence of ADA. These values for CD83 (Left column), CD80 (middle column) and CD86 (right column) were then correlated with patient’s viral load (A), CD4^+^ cell counts (B), CD4^+^ Nadir (C) and CD8^+^ cell counts (D). The Spearman correlation test was applied.
(TIF)

Figure S4 mDCs viability. iDCs, obtained as described in the Materials and Methods, from healthy or HIV-infected donors were cultured during 48 h in presence of a maturating cocktail (mDCs). Cell viability was assessed through DIOC_6_ and propidium Iodide (PI) staining and measured by flow cytometry. In A, contour plots showing the percentage of viable (bright DIOC_6_ and negative propidium iodide staining), apoptotic (low DIOC_6_ and negative propidium iodide staining) and necrotic (low DIOC_6_ and positive propidium iodide staining) populations from a representative healthy or HIV-infected donor are shown. The percentage of viable (B), apoptotic (C) and necrotic (D) DCs from 4 different healthy and 6 HIV-infected donors are shown.
(TIF)
