# Supplementary figures and images for: Adenosine Deaminase Enhances the Immunogenicity of Human Dendritic Cells from Healthy and HIV-Infected Individuals
Source: PLoS One. 2012 Dec 11;7(12):e51287. doi: 10.1371/journal.pone.0051287 (PMC3519778; doi:10.1371/journal.pone.0051287)

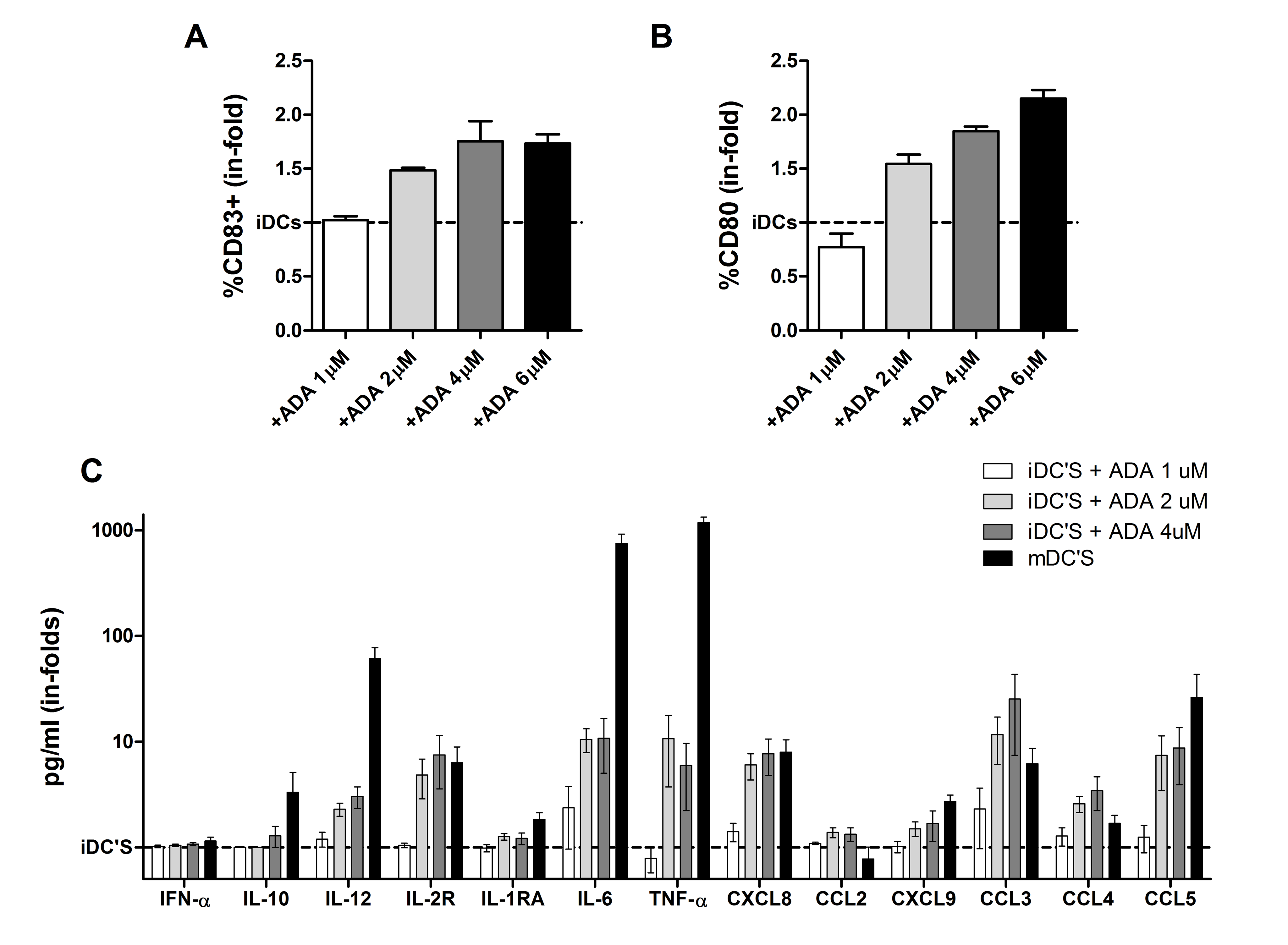

Supplement: Figure S1 — ADA dose-response in CD80/83 expression and cytokine secretion. iDCs, obtained as indicated in the Materials and Methods, were cultured for 48 h in medium in the absence (iDC) or in the presence of 1, 2, 4 or 6 µM ADA (+ADA) and the expression of CD83 (A) or CD80 (B) was addressed by flow cytometry in the DCs gate. Bars indicating the mean ± SEM of 2 independent experiments are shown. In (C) the indicated cytokines and chemokines were determined in the supernatant from iDCs cultured in the absence (iDCs) or in the presence of 1, 2, 4 µM ADA or in the presence of the maturating cocktail (mDCs). Bars indicating the mean and SEM of 4 different experiments are shown. Values are expressed as the ratio (in-fold) of CD83, CD80 or cytokine/chemokine levels obtained in the presence of ADA or mDCs versus levels obtained in the absence of ADA (iDCs, the reference value of 1 is represented by a dotted line). (TIF) [file pone.0051287.s001.tif]

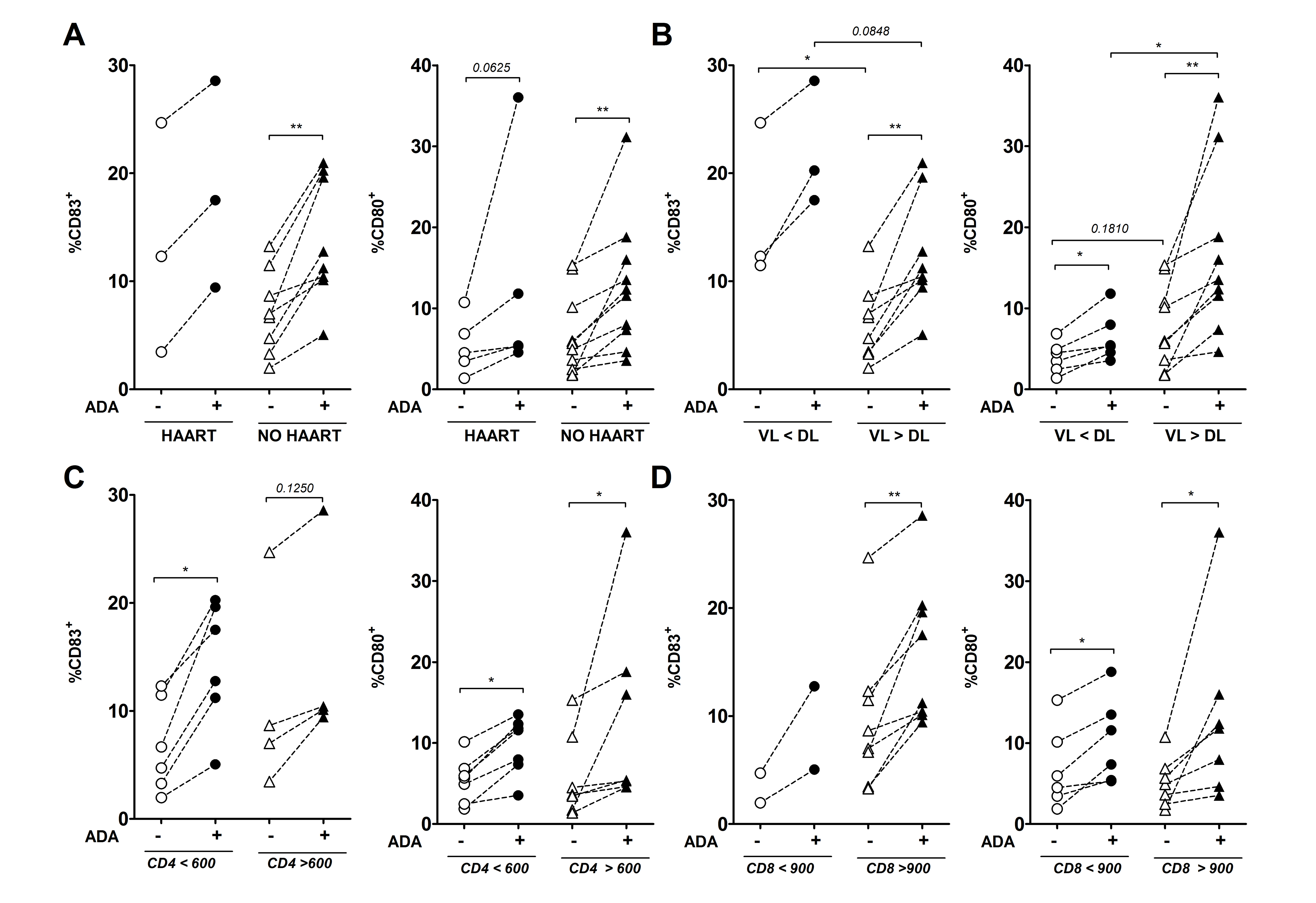

Supplement: Figure S2 — CD80 and CD83 expression on iDCs from HIV-infected subjects according to different clinical parameters. iDCs from HIV-infected subjects were cultured for 48 h in medium in the absence (−ADA) or in the presence of 2 µM ADA (+ADA) and the expression of CD83 and CD80 was addressed by flow cytometry. Patients were separated according to receiving HAART or not (A), having undetectable (<DL) or detectable viral load (>DL) (B), being above or below 600 CD4+/µL (C) or above or below 900 CD8+/µL (D).*P<0.05, **P<0.01. (TIF) [file pone.0051287.s002.tif]

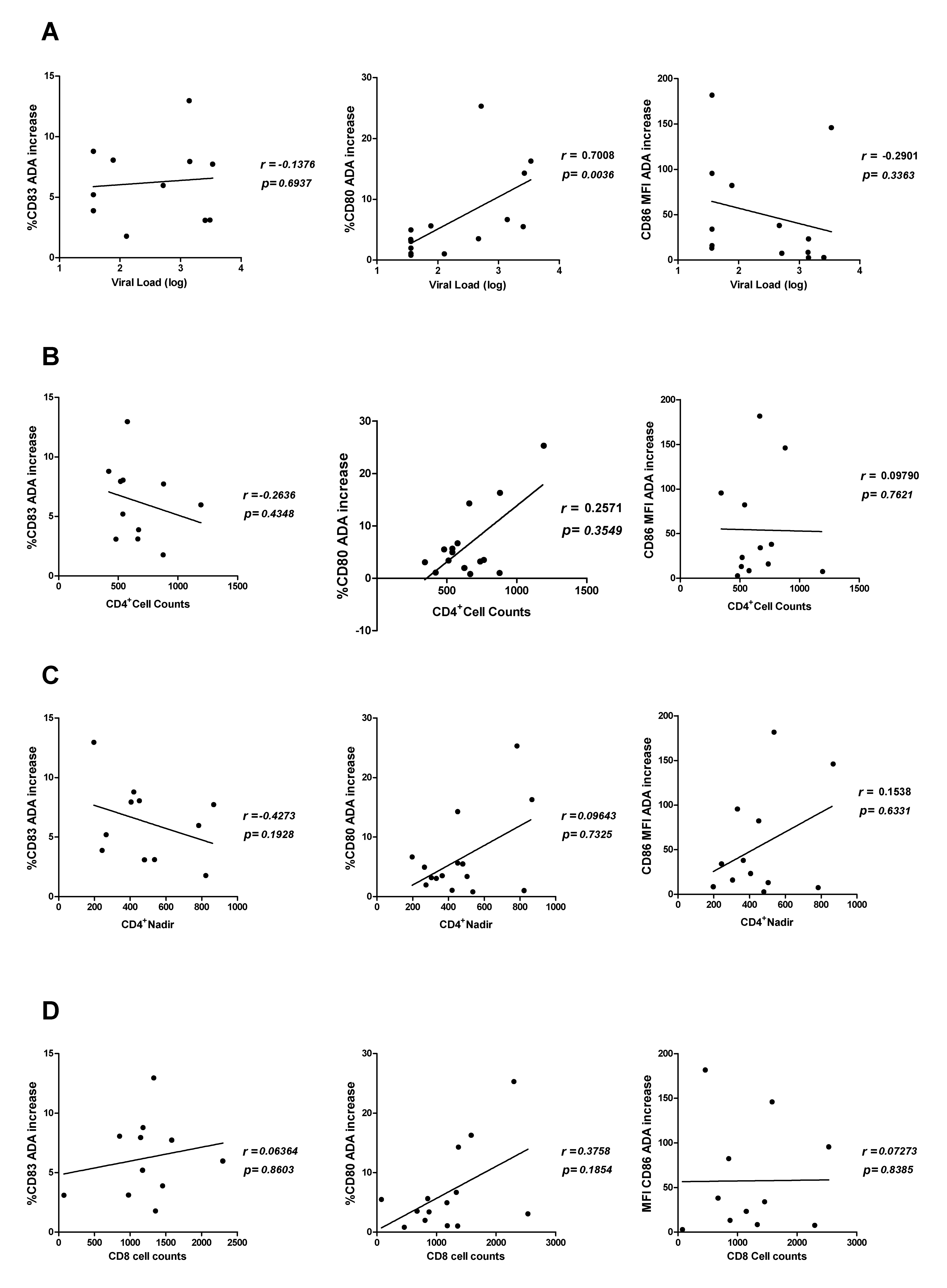

Supplement: Figure S3 — Correlation of ADA effect on CD83, CD80 and CD86 expression on iDCs from HIV subjects with different clinical parameters. iDCs from HIV-infected subjects were cultured for 48 h in medium in the absence (−ADA) or in the presence of 2 µM ADA (+ADA) and the expression of CD83, CD80 and CD86 was addressed by flow cytometry. The % of ADA increase on the expression of each marker was obtained by subtracting the percentage of expression in the absence of ADA from the the percentage of expression in the presence of ADA. These values for CD83 (Left column), CD80 (middle column) and CD86 (right column) were then correlated with patient’s viral load (A), CD4+ cell counts (B), CD4+ Nadir (C) and CD8+ cell counts (D). The Spearman correlation test was applied. (TIF) [file pone.0051287.s003.tif]

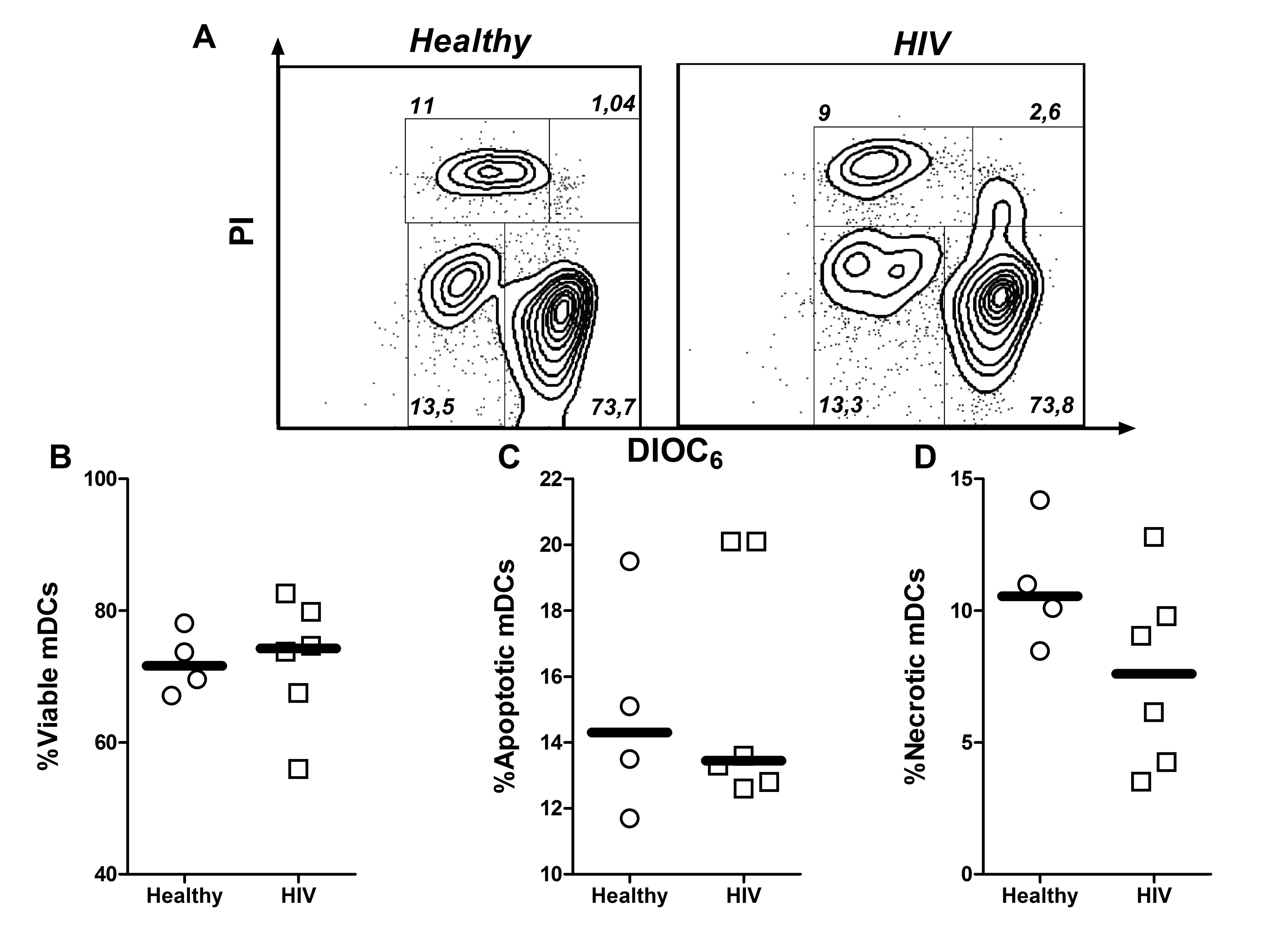

Supplement: Figure S4 — mDCs viability. iDCs, obtained as described in the Materials and Methods, from healthy or HIV-infected donors were cultured during 48 h in presence of a maturating cocktail (mDCs). Cell viability was assessed through DIOC6 and propidium Iodide (PI) staining and measured by flow cytometry. In A, contour plots showing the percentage of viable (bright DIOC6 and negative propidium iodide staining), apoptotic (low DIOC6 and negative propidium iodide staining) and necrotic (low DIOC6 and positive propidium iodide staining) populations from a representative healthy or HIV-infected donor are shown. The percentage of viable (B), apoptotic (C) and necrotic (D) DCs from 4 different healthy and 6 HIV-infected donors are shown. (TIF) [file pone.0051287.s004.tif]
